# Nasal profile changes after LeFort I osteotomy (maxillary advancement) with and without ANS reduction in class III skeletal patients: a study protocol for a randomized clinical trial

**DOI:** 10.1186/s13063-024-08162-1

**Published:** 2024-05-27

**Authors:** Mehdi Sezavar, Hossein Rastegar Moghaddamshaldoozi, Afshin Haraji, Nima Ghanipour, Salar Chaychi Salmasi

**Affiliations:** 1 Department of Oral and Maxillofacial Surgery, Dental Faculty, Tehran Medical Sciences, Islamic Azad University, Tehran, Iran; 2 Tehran Medical Sciences, Islamic Azad University, Tehran, Iran

**Keywords:** Labial soft tissue change, Maxillary advancement surgery, Nasolabial angel changes, Orthognathic surgery

## Abstract

**Background:**

Dentofacial malformation is a common condition that affects a significant portion of the population, resulting in functional and aesthetic defects. Orthognathic surgeries, such as LeFort I osteotomy, are performed to correct these abnormalities. However, the impact of these surgeries on nasal profile changes remains unclear. Additionally, the role of anterior nasal spine (ANS) reduction in maxillary advancement surgeries of 3–5 mm range is yet to be determined. This study aims to investigate the effect of ANS reduction on soft tissue profile changes following LeFort I osteotomy with a maxillary advancement range of 3–5 mm in class III skeletal patients. The hypothesis is that the changes in nasolabial angle and upper lip length will not significantly differ between patients who undergo LeFort I osteotomy with and without ANS reduction.

**Method and design:**

This study is designed as a randomized controlled trial. A total of 26 class III skeletal patients with maxillofacial abnormalities will be recruited from the maxillofacial clinic of Bu-Ali and Farahikhtegan Hospitals in Tehran, Iran. Patients meeting the inclusion criteria will be randomly assigned to two groups: one group will undergo LeFort I osteotomy with ANS reduction, and the other group will undergo LeFort I osteotomy without ANS reduction. The soft tissue profile changes, specifically the nasolabial angle and upper lip length, will be evaluated and compared between the two groups.

**Discussion:**

Achieving facial harmony through orthognathic surgery requires careful planning and consideration of the impact on surrounding soft tissue. The primary objective is to predict and plan for the effects on the nasolabial region. LeFort I osteotomy is a common procedure used to correct dentofacial deformities, particularly in class III patients. Maxillary advancement during this surgery can lead to changes in nasal tip position, width, and rotation, potentially due to repositioning of the anterior nasal spine and soft tissue dissection. In this study, soft tissue changes will be assessed in non-growing class III patients using cephalometric radiographs. The impact of reducing the anterior nasal spine (ANS) on nasal profile changes will be investigated for maxillary advancements of 3–5 mm. Objective measurements and patient-reported outcomes will be evaluated to gain insights into the aesthetic outcomes of orthognathic surgery. The findings will provide valuable guidance for treatment decisions and alternative options based on expected nasal profile changes.

**Trial registration:**

This project was registered at The Iranian Registry of Clinical Trials (Identifier No. IRCT20210928052625N1, Website: https://www.irct.ir/trial/59171) and Open Science Framework (OSF) (Registration https://doi.org/10.17605/OSF.IO/X3HD4). 2021-06-09.

**Supplementary Information:**

The online version contains supplementary material available at 10.1186/s13063-024-08162-1.

## Introduction

Dentofacial malformation is the deviation of facial skeletal development from its correct and normal process, which manifests itself with changes in appearance, malocclusion, and functional problems [1]. This anomaly affects approximately 20% of the population and includes a different spectrum of functional and aesthetic defects. Dentofacial deformity can be limited to the maxilla or mandible or spread to more structures. It can also be unilateral or bilateral and in a different amount in terms of longitudinal, transverse, or vertical dimensions [2]. Orthognathic surgeries are used to treat these abnormalities. The time to perform these surgeries is after the end of growth and will be performed if orthodontic treatment alone is not enough to solve these abnormalities. Orthognathic surgery along with orthodontic treatment will result in more stable outcomes in terms of improving facial appearance and function [3]. The success of the treatment depends on the diagnosis and the appropriate treatment plan and the skill of the surgeon, and the change in the hard tissues changes the shape of the soft tissue [4]. The technique and knowledge of orthognathic surgeries have made great progress in recent years, but our understanding and awareness in terms of predicting soft tissue changes remains limited [5, 6]. The most changes of the soft tissue after LeFort 1 osteotomy surgery are related to nose type, nasolabial angle, alar base width and upper lip position, and height [7]. Some of these soft tissue changes lead to better cosmetic results and are therefore desirable, and therefore we allow them to occur after surgery. For example, a patient who has a small interalar space before LeFort 1 osteotomy surgery will benefit from maxilla advancement. Because this surgery will increase this distance, and for this patient it is considered a favorable result in terms of aesthetics.

On the other hand, by predicting changes that may have an adverse effect on beauty, these effects can be reduced by taking auxiliary measures during surgery, or by informing the patient before surgery, with the help of secondary surgeries to fix these problems [8]. The nose is an important part of facial beauty. LeFort 1 osteotomy and maxillary movements affect the position and shape of the nose. In some studies, widening of the base of the nose following LeFort 1 osteotomy has been reported [9]. In general, changes in the nose have remained contradictory and unclear in studies. In any case, it is necessary for the surgeon and the patient to be aware of possible soft tissue changes following surgery and to know that they may need secondary corrective surgeries to achieve the desired aesthetic results after surgery [10]. The existing literature review indicates that there is a significant debate surrounding the alterations in nasolabial soft tissue following maxillary advancement surgery so, there is not a consensus idea on the alterations of LeFort I Maxillary Surgery in nasolabial soft tissue. Based on the Surgeons’ experience in this Study, we have ANS reduction for cases above 5 mm of maxillary advancement, and below 3 mm does not require ANS reduction [11]. Still, in cases where we have maxillary advancement between 3 and 5 mm, some surgeons tend to reduce ANS and some do not, so the purpose of our study is to find out that which method is better in the range of 3–5 mm.

### Objective and hypothesis

The major goal of this double arm parallel randomized clinical trial is to see how reducing ANS affects the soft tissue profile after maxillary advancement surgery with an advancement range of 3–5 mm. The null hypothesis is that the changes of nasolabial angle and upper lip length in skeletal class III patients of the study after LeFort 1 osteotomy (maxillary advancement) with and without ANS reduction are not significantly different.

## Methods

### Overview

**Fig. 1:**
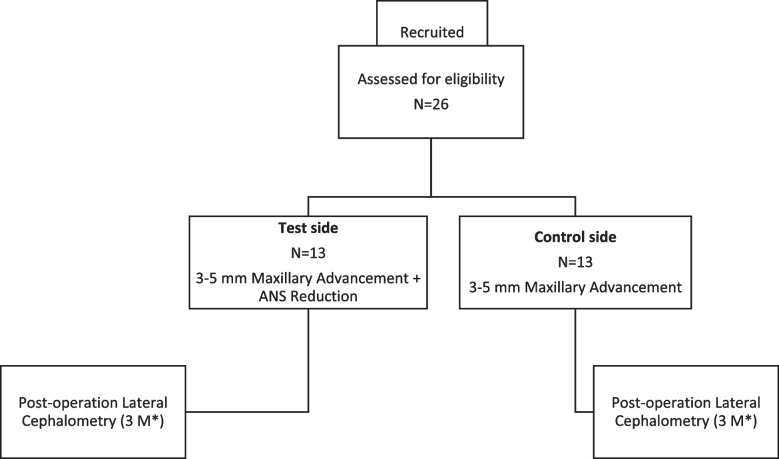
Flow chart of the treatment process. “*” symbol indicates the following: M, month

This research is planned as a randomized controlled trial. The goal is to enroll 26 class III skeletal patients from the patients who refer to the maxillofacial clinic of Bu-Ali and Farahikhtegan Hospitals in Tehran, Iran, who suffer from maxillofacial abnormalities. We will have 2 groups: (1) control group (without ANS reduction) and (2) intervention group (with ANS reduction). This project was registered at The Iranian Registry of Clinical Trials (Identifier No. IRCT20210928052625N1, Website: https://www.irct.ir/trial/59171) and Open Science Framework (OSF) (Registration 10.17605/OSF.IO/X3HD4). Recruiting and treatment methodology of this study is confirmed by the Research Ethics Committee of the Islamic Azad University Dental Branch, Tehran, Iran (IR.IAU.DENTAL.REC.1400.035). Each participant’s overall health will be assessed by researchers before creating a treatment plan (Fig. 1 and Table 1).

**Table 1:** SPIRIT figure

	**Study period**			
	**Enrolment**	**Allocation**		
	**Prior to allocation**	**Month** **0**	**Month** **3**	**Month** **4**
Case selection Allocation Hygiene education	*	*		
Orthognathic surgery and pre-operative lateral cephalometry		*	*	*
3 month recovering time				*
Post-operative lateral cephalometry			*	*

Inclusion criteria:

1. The amount of maxillary advancement should be 3–5 mm.
2. Participants must be between 20 and 50 years to enter the study.

Exclusion criteria:

1. Presence of Jaw surgery in the medical history
2. Presence of rhinoplasty or any other nose surgeries in medical history
3. Need of maxillary advancement below 3 mm
4. Need of maxillary advancement more than 5 mm
5. Any systemic or local diseases that may interfere with bone repair (including hypothyroidism, osteoarthritis, hyperparathyroidism, diabetes, and osteoporosis)
6. Using bisphosphonates
7. Pregnancy
8. Smoking or drug addiction
9. Patient undergoing radiotherapy
10. Presence of syndromes or cleft palates [3, 12]

Patients who with any of these requirements will be excluded from the research.

### Recruitment

Patients with skeletal class III pattern, with maxillofacial disorders, are eligible for a maxillary advancement surgery. Between 2023 and 2024, patients are sent to the Oral and Maxillofacial Surgery Department, Bu-Ali and Farhikhtegan Hospitals, Tehran, Iran. Patients who meet the criteria will receive a written consent form that clearly explains the details of the study in simple language. The researchers will also verbally explain the background of the research, how it will be conducted, what participation involves, any potential risks, and the rules regarding confidentiality. Participants have the right to ask questions and receive all the necessary information about the study. Prior to enrolling in the research, each patient must sign a consent form to confirm their understanding of the study’s objectives. They will then proceed to join the research after expressing their willingness to participate.

## Allocation and randomization

### Randomization

Every participant will receive a unique numerical code, which will be enclosed within a securely sealed envelope. The envelopes will remain unopened until the procedure is conducted by external investigators. As a result, the participants will be divided into two groups: group 1 (with ANS reduction) and group 2 (without ANS reduction). An independent investigator will utilize a web-based randomization service available at www.randomization.com to generate a randomized sequence. The envelopes are opened during the surgery by surgical assistant. Until that moment, the result of the allocation process in each sample is not known.

### Blinding

The operator cannot be blinded, but the treatment assignment will be kept concealed from the data assessor. Additionally, all patients will be unaware of their group allocation. Operators will not have access to statistical assessment tools to maintain blinding. Similarly, statisticians will remain unaware of the treatments and group assignments.

### Intervention

For these patients, a standard lateral cephalometry (the ORTHOPHOS brand device, which has a voltage of 200 to 240 volts and takes images with an output of 230 volts) is prepared before the operation, and the points and angles of the study will be analyzed using a gauge and a calibrated protractor [5, 10, 13, 14]. On the day of the operation, two sets of surgery are prepared according to the two types of study interventions, and at that time, the corresponding envelope is opened according to the order of referral and the patient’s assigned code, and the type of intervention is determined. We have 2 groups: the control group will not have any ANS reduction, and the test group will have ANS reduction. For patients undergoing surgery, after the patient is anesthetized by the anesthesia service, the routine preparation and the necessary injections will be performed in the surgical areas. Then, we will start the incision with the surgical blade no. 15 in the depth of the buccal vestibule of the upper jaw from the alignment of upper right first molar (16) to upper left first molar (26), the flap is pushed aside, and the periosteum will be separated from the bone in order to have the access to the bone for LeFort I incision. Then, the osteotomy lines are drawn and cut with a saw, and the maxilla will be separated from the base of the nose after the down fracture; then, the maxilla is placed in a new and appropriate position (advancement 3–5 mm) and by two “L” plates with 4 holes, and two straight plates with 4 holes will be fixed. If there is a need for ANS reduction, the ANS is completely removed by a large-sized round carbide bur. The surgical process continues, and after the surgery, we will follow-up the patient for 3 months, and after 3 months, we will request a post-op radiography to collect final data [15].

The second lateral cephalometry will be requested from the same radiology center in the same position as the previous one. The desired data for the research work will be measured by the same person with the same equipment; it should be noted that all the cephalometric measurements (the baseline and follow-up radiographs) will be measured twice, and the average is recorded as the desired number of that variable; then, the obtained results will be subjected to statistical analysis (Fig. 1). The patients will be excluded from the trial if they get pregnant, leave their orthodontic treatment half-finished, or leave the study at their own will.

### Outcomes

An oral and maxillofacial radiologist will assess the nasolabial angle and upper lip length using a lateral cephalometry before the surgery. Three months following surgery, a second lateral cephalometry will be performed. With the same basic evaluation methods, nasolabial angle and upper lip length will be assessed.

### Sample size

According to the results of the study by Mahsoub R, et al., using the Advanced Repeated Measures Anova Power Analysis option of the Pass11 software, considering *α* and *β* = 0.05 for both within-between subject variables equal to 0.2, the repeated factor with two repetitions and the effect size equal to 0.5 and the two-way between subject factor with an effect size equal to 0.6, the minimum sample size required for each of the two study groups was calculated as 13 samples [16].

### Data collection and management

All participants will be allocated an individual trial identification number during the study. Data collection is limited to authorized members of the research team. These team members are required to maintain confidentiality and utilize the data solely for scientific purposes. The information is securely stored both electronically and in hard copy format. Investigators are provided with unique login credentials to access the data on the secure platform, ensuring privacy. To prevent bias, an independent investigator will perform the statistical analysis.

### Training and calibration

The surgical process will be performed by one specialist (M.S) with over 15 years of professional working experience. He is also an expert in the field of orthognathic surgery. An independent investigator will do the measurements before the surgery and after that. The research team consists of an oral and maxillofacial surgery resident and a final-year dental student, both of whom have a minimum of 4 years of clinical experience and have received training in accuracy and reproducibility to ensure compliance. A certified researcher with extensive years of expertise will be responsible for collecting clinical data, patient testimonials, and information on complications to maintain consistency.

### Statistical analysis

To assess the consistency of descriptive data, Cohen’s *k* statistic will be employed. Descriptive statistics, such as frequency counts (both absolute and relative) and metric data (such as arithmetic mean, standard deviation, and median), serve as examples of the types of statistics used. The consistency between the two examiners for continuous data, such as clinical parameters, will be assessed using the intraclass correlation coefficient. Enumerated data will be presented as percentages, while measured data will be reported as mean standard deviation or median (quartile spacing). We will assess the effects of differences in measurement time between the two study groups at baseline and follow-up by conducting an analysis of variance (ANOVA), considering the type of intervention as a factor. All statistical analyses will be conducted using SPSS 25.0. The significance threshold is set at 0.05.

### Missing data

When determining the sample size for the examination, the potential for participants to become untraceable is considered and factored into the calculations. Furthermore, any missing data (such as loss to follow-up, death, withdrawal, etc.) will be addressed using the multiple imputations approach to ensure completeness.

## Ethical consideration

### Ethical approval

The recruitment and treatment procedures for this study were approved by the Research Ethics Committees, Islamic Azad University-Dental Branch, Tehran, Iran (IR.IAU.DENTAL.REC.1400.035). Eligible participants will receive detailed information about the trial’s consent requirements. Individuals who do not provide consent will not be included in the final analysis.

### Withdrawal

All patients will be given all the information they need about the study before any treatment begins. Patients can leave the study at any time for any reason. All patients will receive the best care possible, whether or not they participate in the study.

### Publication of results

The results of the study will be published in a scientific journal, and the analysis will be posted online for everyone to see.

## Oversight

### Trial management

The current clinical study is being led by the Oral and Maxillofacial Department, Dental Faculty, Tehran Medical Sciences, Islamic Azad University, Tehran, Iran. The research team will receive support and assistance from this coordinating center in various aspects of the study, such as trial design, quality assurance, data analysis, dissemination, and study management.

### Steering committee

The trial steering group consists of multiple patients, public representatives, two independent doctors, and a statistical expert who have been recruited to provide guidance and oversight throughout the study. The committee members will convene with the study group every 2 months and will not be directly involved in the operational aspects of the trial. Their responsibilities include offering recommendations to the research team regarding the trial’s implementation, data management, monitoring, recruitment, and follow-up methods. They will also be responsible for analyzing the results and tracking the progress of the trial to make sure it is being done according to the plan.

### Data monitoring

The trial’s advancement, any adverse events, and data quality will be overseen by an independent data monitoring committee (DMC). This committee consists of members from the Ethics Committee of the Dental Faculty, Tehran Medical Sciences, Islamic Azad University, Tehran, Iran, who are not associated with the trial investigators, research team, or sponsors. The research team will regularly meet with an auditor appointed by the DMC every 2 months to monitor the study’s progress and conduct an initial analysis. The DMC has the authority to halt the trial if any injuries or risks are identified based on interim findings.

### Harms

There will be no significant injury to research participants, and the surgery method has a minimal probability of failure. The same surgeon will do all of the operations. To reduce the danger of injury, all required precautions will be followed. If something goes wrong, the participants will have a backup restoration plan in place. If a surgical failure occurs together with unanticipated side effects or major adverse events, the DMC will be notified very once.

### Audits

The DMC will have an inspector verify the incoming data every 3 months, independent of the investigators. Each electronic case report form will be checked by the inspector for completion.

## Discussion

Achieving facial harmony through the correction of dentofacial anomalies requires careful and efficient planning and execution of orthognathic surgery. It is crucial to recognize that each action taken to achieve proper dental occlusion has an impact on the surrounding soft tissue. The primary aim of orthognathic surgery is to enhance both facial and dental aesthetics. This is achieved through a combination of orthodontic and surgical interventions to address facial deformities. For a majority of patients, the key driving factor behind seeking this treatment is the desire for improved soft tissue appearance [11]. First, we have to accurately predict the effects of each step on the soft tissue, particularly in the nasolabial region, and then plan the surgical approach accordingly [15]. Orthognathic surgery is performed to correct dentofacial deformities and enhance the functional relationship between the upper and lower jaws, leading to improved facial harmony. One common procedure used for this purpose is LeFort I osteotomy, which involves repositioning the maxilla in all three spatial planes to address various deformities. In this particular study, LeFort I osteotomy is utilized to achieve maxillary advancement in patients with class III deformities [3, 17]. Maxillary superior repositioning and advancement during orthognathic surgery can result in changes to the nasal tip position and the width of the nose as well as an upward rotation of the nasal tip and augmentation of the alar base. These changes are observed regardless of the extent of maxillary advancement or anterior/posterior impaction. One possible explanation for these alterations is that the repositioning of the anterior nasal spine and the dissection of soft tissues during surgery can lead to a partial loss of the original nasal measurements, resulting in subtle distortions [3].

In the present study, we will assess soft tissue changes in the middle and lower third of the face in a group of twenty-six non-growing patients with class III malocclusion. Cephalometric radiographs will be used to measure linear and angular parameters, allowing us to evaluate the alterations in soft tissue following maxillary advancement. It is worth noting that a study conducted by Nagori et al. in 2017 reported a reduction in nasal prominence after maxillary advancement, which contradicts the findings of other studies on the subject [11]. This randomized clinical research aims to investigate the impact of reducing the anterior nasal spine (ANS) on nasal profile changes following orthognathic surgeries that require a 3–5-mm advancement of the maxilla. The study will evaluate both objective data and patient-reported outcomes to provide valuable insights into the effects of ANS reduction on the aesthetic outcomes of orthognathic surgery. The findings of this research will be beneficial in guiding treatment decisions for patients who are considering orthognathic surgery, offering alternative options based on the expected nasal profile changes.

## Challenges and limitations

One of the challenges that may occur during the LeFort I osteotomy is the incidence of hematoma and edema. Nasofrontal angle may change due to errors during surgery. It is also possible to create a depression in the sub-nasal area due to excessive removal of the ANS.

## Trial status

Patient recruitment is currently in progress at the time of manuscript submission (date of submission). The recruitment process will be finished until the end of December 31, 2023, and it is anticipated to be finalized by April 01, 2024.

### Supplementary Information

Supplementary Material 1.

## Abbreviations

ANS: Anterior nasal spine
DMC: Data monitoring committee
